# Control of a New Financial Risk Contagion Dynamic Model Based on Finite-Time Disturbance

**DOI:** 10.3390/e26120999

**Published:** 2024-11-21

**Authors:** Yifeng Wei, Chengrong Xie, Xia Qing, Yuhua Xu

**Affiliations:** 1 School of Economy and Management, Hanjiang Normal University, Shiyan 442000, China; wuhanwyf@163.com; 2 School of Mathematics, Nanjing Audit University, Nanjing 211815, China; xcr0603@163.com; 3 School of Economics, Guangzhou City University of Technology, Guangzhou 510800, China; 4 Research Base of Carbon Neutral Finance for Guangdong-Hong Kong-Macao, Guangzhou 510800, China; 5 School of Statistics and Data Science, Nanjing Audit University, Nanjing 211815, China; yuhuaxu2004@163.com

**Keywords:** chaos, bifurcation, finite-time synchronization

## Abstract

With the widespread application of chaotic systems in many fields, research on chaotic systems is becoming increasingly in-depth. This article first proposes a new dynamic model of financial risk contagion based on financial principles and discusses some basic dynamic characteristics of the new chaotic system, such as equilibrium points, dissipativity, Poincaré diagrams, bifurcation diagrams, etc. Secondly, with the consideration of privacy during data transmission, the method was designed to protect the privacy of controlled systems in finite time based on perturbation. A controller designed for finite time was developed based on Lyapunov stability principles, which achieves system synchronization within a finite time and protects the privacy of the controlled system. The effectiveness was also verified by numerical simulations.

## 1. Introduction

A financial chaotic system is an important research direction in the field of finance, which involves nonlinear dynamics, complexity science, and financial engineering. In recent years, driven by the growth of financial technology, the research and application of financial chaotic systems have made significant advancements. For example, in 1994, Xu et al. gave the economic meaning of a chaos model and the definition of economic chaos by discussing the chaos phenomenon of economic models [1]. Yang Zihui et al. investigated the inter-market transmission of economic policy uncertainty in conjunction with systemic financial risk from the perspective of nonlinear complex systems [2]. Chen et al. conducted an analysis of the bifurcation and chaotic dynamics associated with credit risk contagion using the Fitzhugh–Nagumo system [3]. Yuan et al. established the minimum cost-sensitive error probability for predicting extreme events, along with its information-theoretic lower and upper limits by means of a chaotic system [4]. Wu et al. discussed double-well stochastic resonance for a class of three-dimensional financial systems [5]. Furthermore, scholars have studied the stability, bifurcation behavior, and chaotic characteristics of financial chaotic systems through mathematical modeling and dynamic analysis. For example, Wen et al. explored the evolution of complexity within chaotic financial systems by means of fractional calculus [6]. Ma et al. scrutinized the application and research of fractional differential equations in the dynamic analysis of chaotic financial systems within supply chains [7]. Shi et al. conducted an investigation into chaos, Hopf bifurcation, and the control mechanisms of a fractional-order delayed financial system [8]. Recently, some financial risk models have also been discussed. For example, Luo et al. discussed multi-scale financial risk contagion among international stock markets [9]. Akhtaruzzaman et al. discussed financial contagion during the COVID-19 crisis [10]. Koliai et al. discussed an EVT-pair-copulas approach for financial stress tests [11]. As financial data are the discrete output produced by the complex system of the financial market in the evolution process, many of the change rules of these data show obvious nonlinear characteristics, such that people’s research on financial systems is no longer limited to using mathematical statistics and other traditional methods to mine the characteristics of the data. Moreover, the economic relationship hidden in financial data is an evolutionary process. Once the dynamic mechanism of this evolution is determined, it is of great significance to understand and analyze the evolutionary behavior of the data. Therefore, a micro-description of the dynamic characteristics of financial data based on complex system theory will provide a new perspective for financial data analysis.

Many control strategies have been proposed to manage and regulate financial chaotic systems, including equilibrium control, adaptive control, and synchronous control. The aim of these approaches was to reduce financial risk by transforming the system from chaos to order through external intervention. For example, Zhang et al. probed into the stabilization of a 4D financial system subject to disturbances and uncertainties through a control method based on UDE [12]. Huang et al. carried out an exhaustive exploration of linear control approaches with the aim of attaining synchronization in a fractional-order time-delayed chaotic financial system [13]. Huang et al. examined an active control strategy aimed at achieving synchronization and anti-synchronization in a fractional chaotic financial system [14]. Gong et al. investigated chaotic behavior and adaptive synchronization in a specific class of fractional-order financial systems [15].

For the past few years, there has been an increasing focus on integrating concepts of privacy protection into the multi-agent consistency problem [16]. At present, some authors have successfully applied the concept of privacy protection to the consistency research of multi-agent systems [17]. In order to expand the research field, Duan proposed a privacy protection mechanism in [18], which was applied to the multi-agent maximum consistency algorithm. It calculates the probability that the agent with the largest state is recognized by the neighbor. Huang et al. first studied differential privacy in the context of multi-agent consistency [19]. As shown in [20], the author discusses the consistency algorithm for heterogeneous multi-agent systems in continuous time based on differential privacy. In [21], the authors propose a conceptually different framework for the privacy protection of multi-agent dynamic initial states. A wide continuous finite-time-varying transformation form is constructed in the interval to realize the privacy protection of multi-agents [22]. The literature [23] addresses the finite-time synchronization of complex networks using privacy protection methods.

As far as we know, to date, privacy protection has received little attention based on a dynamic model of financial risk contagion with finite-time perturbation. Encouraged by the above discussion, our brief results try to address the following two issues: (i) According to financial theory, the greater the contagion effect, the more difficult to control systemic risk; that is, the contagion effect is more destructive. A new financial risk contagion model is proposed. (ii) Based on the form of perturbation, the finite-time control method of the financial risk model is designed, and the privacy protection of financial risk controlled model is realized in the process of synchronization.

This paper is structured as follows: Section 2 presents the new chaotic system and discusses several fundamental dynamic features. Section 3 introduces the necessary conditions for finite-time synchronization in chaotic systems. The conclusions are outlined in Section 4.

## 2. A Single Equilibrium New Chaotic System

In [24], the authors give the following financial risk contagion dynamic model:

(1) $$\left\{\begin{array}{c} \dot{x}=yz-ax\\ \dot{y}=xz-by\\ \dot{z}=cz-xy \end{array}\right.$$

where *x* represents the overall risk value of the system that is affected by both external and internal shocks during the initial stage of phase *i*; *y* reflects the total risk value of the system arising from contagion effects in the second stage of phase *i*; and *z* denotes the control value for system risk in the third stage of phase *i*. When the parameters are a=5, b=9, and c=1, system (1) is chaotic.

Based on the theory of finance, the greater the contagion effect, the more difficult it is to control systemic risk, that is, the more destructive the contagion effect. Therefore, we obtain the following new dynamic model of financial risk contagion:

(2) $$\left\{\begin{array}{c} \dot{x}=yz-ax\\ \dot{y}=xz-by\\ \dot{z}=cz-dy-xy \end{array}\right.$$

**Figure 1:**
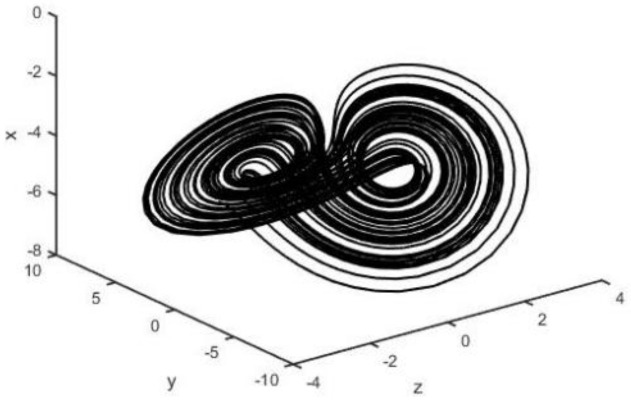
System (2) in x−y−z plane.

**Figure 2:**
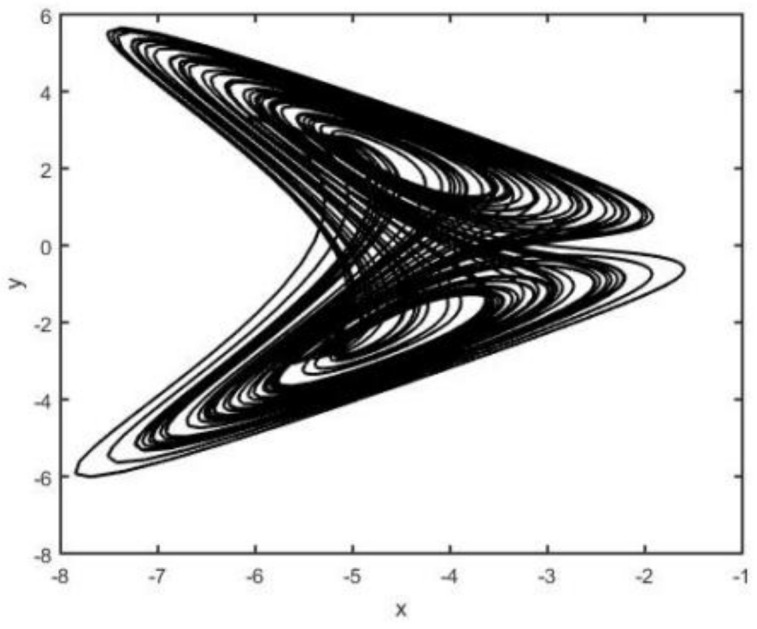
System (2) in x−y plane.

**Figure 3:**
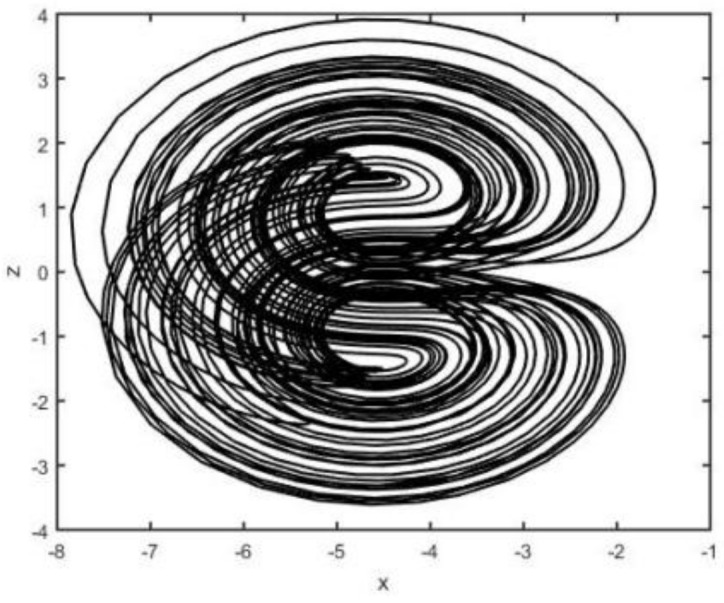
System (2) in x−z plane.

**Figure 4:**
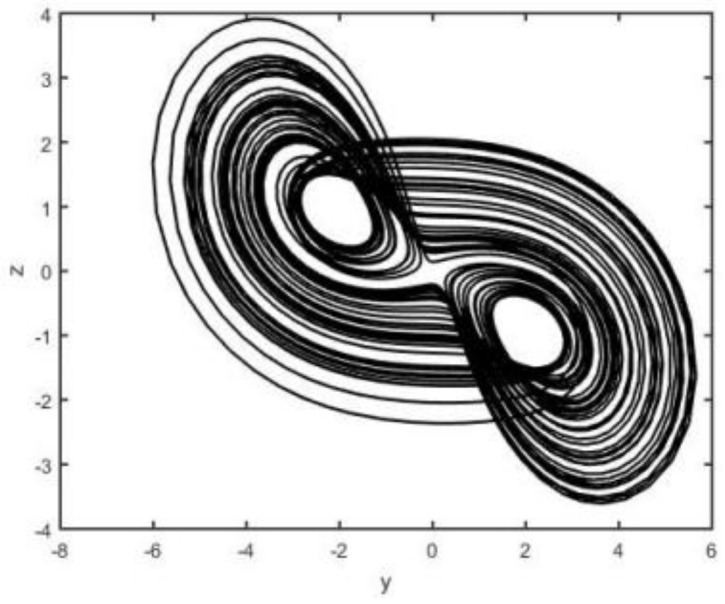
System (2) in y−z plane.

When the parameters a=0.5, b=2, c=0.1, and d=4.5, system (2) is chaotic (see Figure 1, Figure 2, Figure 3 and Figure 4).

**Remark** **1.** *At present, most of the literature on financial dynamic models discusses the evolutionary behavior of discrete models [25,26,27]. However, in the financial system, due to the continuity of economic activities, continuous economic and financial dynamic models may better simulate the real financial system in some situations.*

**Figure 5:**
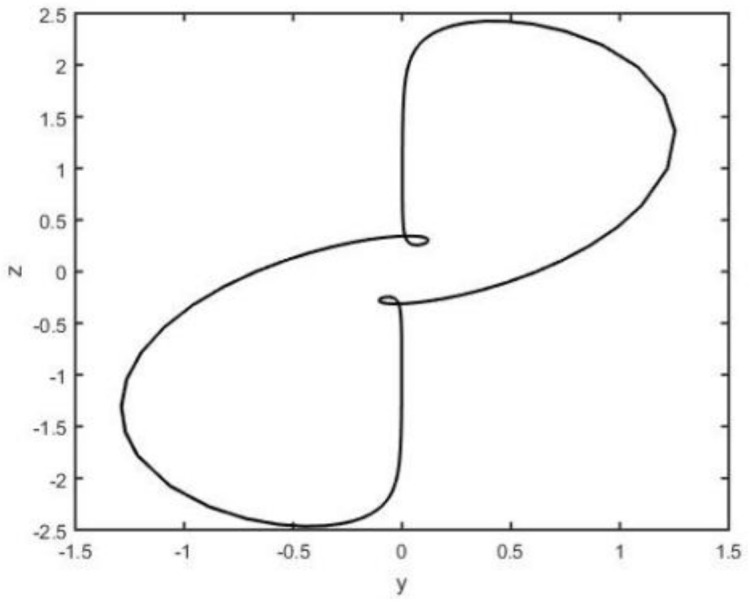
System (3) in y−z plane.

It is clear that when a=0.5, b=2, c=0.1, and d=0, system (2) transitions to system (3). Furthermore, system (3) does not display chaotic behavior for the initial values of (1, 3, 3) (see Figure 5).

(3) $$\left\{\begin{array}{c} \dot{x}=yz-ax\\ \dot{y}=xz-by\\ \dot{z}=cz-xy \end{array}\right.$$

**Remark** **2.** *When y* *= 0, it is obvious that the phase diagram of system (3) is a limit cycle and does not show chaotic characteristics, which means that the contagion effect y* *has a great impact on the system under the current initial value and parameter value. The existence of the system chaos effect increases the uncertainty of risk value in each link of financial risk system management, which will reduce the management efficiency of each link. In addition, because the existence and properties of the limit cycle mainly depend on the structure and parameters of the system, rather than the initial value, in the simulation, the system can also choose different initial values.*

Subsequently, an analysis of the fundamental characteristics of system (2) is conducted.

### 2.1. Equilibria

First, we discuss the equilibria of this nonlinear system. Let

(4) $$\left\{\begin{array}{c} yz-ax=0\\ xz-by=0\\ cz-dy-xy=0 \end{array}\right.$$

If a=0.5, b=2, c=0.1, and d=4.5, the system has three equilibria, which are, respectively, described as follows:

$$E1=\left(0,0,0\right)$$

$$E2=\left(-4.5440,-2.2720,1\right)$$

$$E3=\left(0.0440,-0.0220,-1\right)$$

$$E4=\left(-4.5440,\;2.2720,-1\right)$$

$$E5=\left(0.0440,\;0.0220,\;1\right)$$

For $E1=\left(0,\;0,\;0\right)$, a Jacobian matrix is derived through linearization system (2):

$$J={\left(\begin{array}{ccc} -a & z & y\\ z & -b & x\\ -y & -d-x & c \end{array}\right)}_{E1}.$$

If ${\left|\lambda I-J\right|}_{E1}=0,$ then

$$\lambda_{1}=-0.5,\;\lambda_{2}=0.1,{\;\;\lambda }_{3}=-2.$$

Similarly, we can obtain the corresponding eigenvalues of equilibrium points *E*2–*E*5.

For E2,

$$\lambda_{1}=2.6072,$$

$$\lambda_{2}=–2.5036+1.2549i,$$

$$\lambda_{3}=–2.5036-1.2549i,$$

For E3,

$$\lambda_{1}=-2.4537,$$

$$\lambda_{2}=0.0269+0.2856i,$$

$$\lambda_{3}=0.0269-0.2856i,$$

For E4,

$$\lambda_{1}=-3.0063,$$

$$\lambda_{2}=0.3032+2.6159i,$$

$$\lambda_{3}=0.3032–2.6159i,$$

For E5,

$$\lambda_{1}=-2.4537,$$

$$\lambda_{2}=0.0269+0.2856i,$$

$$\lambda_{3}=0.0269-0.2856i,$$

According to the Lyapunov stability criterion, if the solution of the linearized equation is unstable when the matrix of the linearized equation has at least one positive real part, then the nonlinear system is also unstable. Therefore, *E*1–*E*5 are all unstable.

### 2.2. Dissipativity

For system (2),

$$▽\cdot V=\frac{\partial \dot{x}}{\partial x}+\frac{\partial \dot{y}}{\partial y}+\frac{\partial \dot{z}}{\partial z}=-a-b+c,$$

Given that −a−b+c=−2.4<0, it can be concluded that system (2) is dissipative, and the exponential contraction associated with this system is expressed as exp(−2.4).

### 2.3. Chaotic Behavior of System (2)

**Figure 6:**
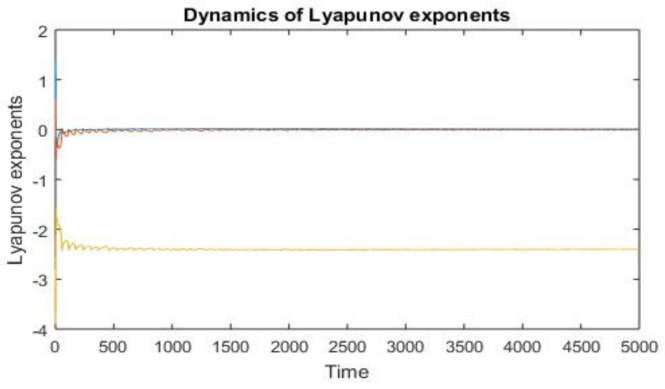
Dynamics of Lyapunov exponents.

When the starting values are assigned as (1, 3, 3), the Lyapunov exponents of system (2) are determined to be $L_{1}=1.3334,$ $L_{2}=0.6080,$ and $L_{3}=-1.5699.$ As system (2) holds two positive Lyapunov exponents, it manifests chaotic behavior (see Figure 6).

In addition,

$$D_{L}=j+\frac{1}{\left|L_{j+1}\right|}\sum_{i=1}^{j}L_{i}=2+\frac{L_{1}+L_{2}}{\left|L_{3}\right|}\;=2+\frac{1.9414}{\left|-1.5699\right|}=3.2366,$$

Thus, the Lyapunov dimension of system (2) is fractional.

**Figure 7:**
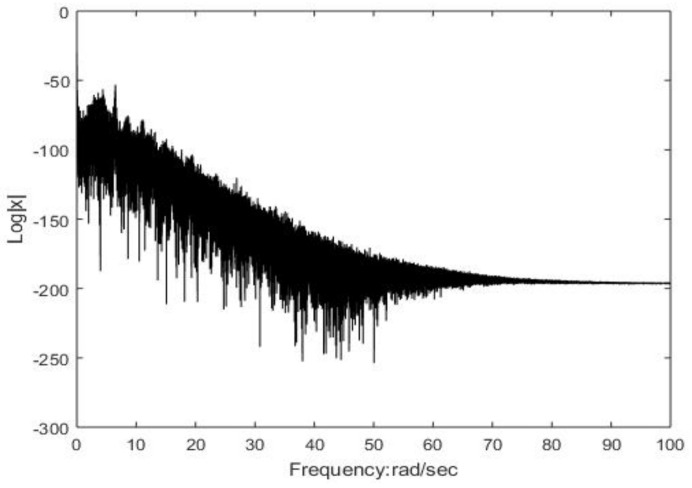
Frequency spectrum diagram.

**Figure 8:**
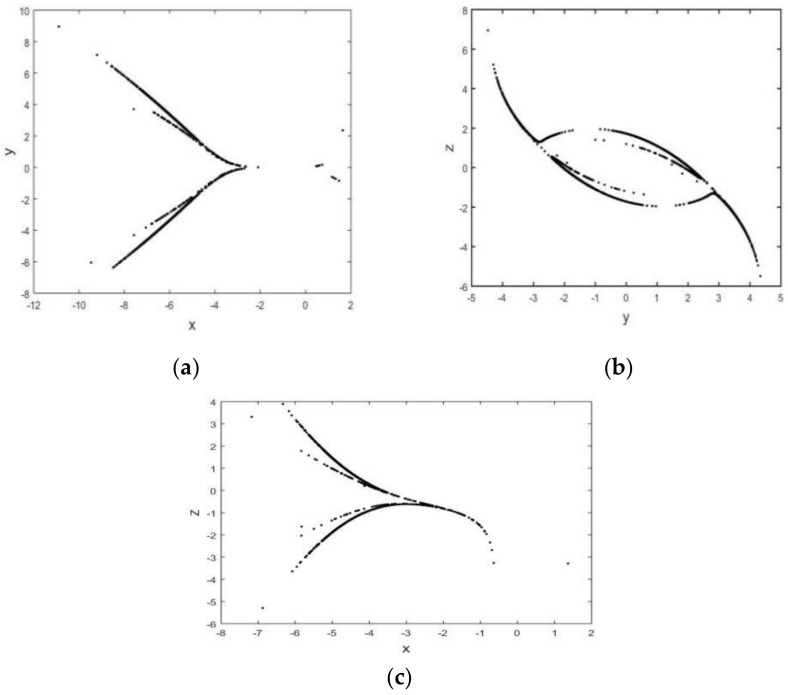
Poincaré diagram for (**a**) z=0, (**b**) x=−5, and (**c**) y=0.5.

**Figure 9:**
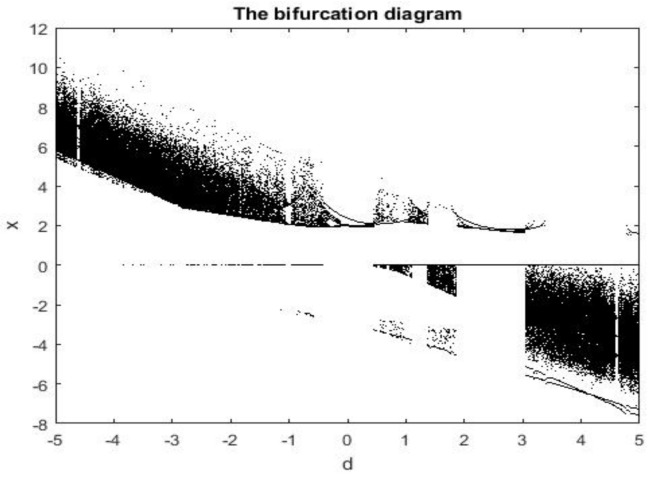
Bifurcation diagram of system (1) for −5≤d≤5.

The continuous broadband attributes of the spectrum of system (2) are depicted in Figure 7. The continuous wideband characteristic explains the behavior of the chaotic system in the frequency domain. Specifically, the spectrum of chaotic signals is not concentrated on a few frequency components but is distributed over a wide frequency range. This wideband feature is an important indicator of chaotic systems, indicating that the behavior of the system is highly complex and difficult to predict. For z=0, x=−5, and y=0.5, Figure 8a–c show Poincaré diagrams of the system at different cross sections, where the blades of the attractor are clearly visible and the blades of the attractor are folded, which leads to the complex dynamic behavior of the system. When the system parameters vary within a defined range, the bifurcation graph can more effectively illustrate the intricate evolutionary behavior of system (2). Figure 9 depicts the bifurcation evolution of system (2) within the range of −5≤d≤5. The bifurcation diagram of chaotic system illustrates the influence of parameter changes on the behavior of the chaotic system and the different possible stable states of the system, which helps to understand various dynamic behaviors in chaotic systems, including periodic behavior, chaotic behavior, and the transition between these behaviors.

## 3. FnT Control of Financial Risk Contagion Dynamic with Finite-Time Perturbation

**Lemma** **1** [20]**.** *For $\varphi_{i}\geq 0,\;i=1,2,\cdots ,n,\;0<\zeta <1,\;\kappa >1,$*

$$\sum_{i=1}^{n}\varphi_{i}^{\zeta }\geq {\left(\sum_{i=1}^{n}\varphi_{i}\right)}^{\zeta },\;\sum_{i=1}^{n}\varphi_{i}^{\kappa }\geq n^{1-\kappa }{\left(\sum_{i=1}^{n}\varphi_{i}\right)}^{\kappa }.$$

**Lemma** **2** [22]**.** *For the system*

(5) $$\dot{x}=f\left(x\left(t\right)\right),\;x\left(0\right)=x_{0},$$

*assume that a continuous and positive-definite function* $\upsilon \left(x\left(t\right)\right)$ *satisfies*

$$\dot{\upsilon }\left(x\right)\leq -\beta_{1}\upsilon^{p}\left(x\right)-\beta_{2}\upsilon \left(x\right),$$

*where* $\beta_{1}>0$*,* $\beta_{2}>0$ *, and* 0<p<1 *. Then, the zero of system (5) is finite-time stable, and*

$$T=\frac{1}{1-p}\frac{1}{\beta_{2}}\ln\frac{\beta_{1}+\beta_{2}\upsilon^{1-p}\left(0\right)}{\beta_{1}}.$$

### 3.1. General Method for Control of Chaotic Systems Based on Finite-Time Disturbance

For the general chaotic system,

(6) $$\dot{x}\left(t\right)=f\left(x\right).$$

In order to enhance the privacy protection during data transmission, we designed the following controlled system with a finite-time perturbation expression:

(7) $$\dot{y}\left(t\right)=f\left(z\left(t\right)\right)+u\left(t\right)$$

(8) $$z\left(t\right)=y\left(t\right)+\frac{1}{l}\left[1+sign\left(T-t\right)\right]\sinh\left(T-t\right),T>0$$

where $u\left(t\right)$ is the controller, l>0, T>0, and $\varphi \left(t\right)=\left[1+sign\left(T-t\right)\right]\left(e^{T-t}-e^{-\left(T-t\right)}\right)$.

**Remark** **3.** *Obviously, $e^{T-t}-e^{-\left(T-t\right)}$* *is a decreasing function.*

**Remark** **4.** *Obviously, in $t\in \left[0,T\right),$ $z\left(t\right)\neq y\left(t\right)$* *and $z\left(t\right)=y\left(t\right)$* *for t≥T,* *so the perturbation term $\frac{1}{l}\left[1+sign\left(T-t\right)\right]\varphi \left(t\right)$* *enhances the privacy protection strength of data $y\left(t\right)$* *for $t\in \left[0,T\right).$*

**Remark** **5.** *According to Remark 4, in order to protect the privacy of controlled system (7) within a finite time, finite-time control technology needs to be designed for during the synchronization between the controlled and driving systems.*

### 3.2. FnT Control of Financial Risk Contagion Dynamic with Finite-Time Disturbance

Consider system (2) to be the driving system, while the controlled system is

(9) $$\left\{\begin{array}{c} {\dot{x}}_{1}=y_{1}z_{1}-ax_{1}+u_{1}\\ {\dot{y}}_{1}=x_{1}z_{1}-by_{1}+u_{2}\\ {\dot{z}}_{1}=cz_{1}-dy_{1}-x_{1}y_{1}+u_{3} \end{array}\right.$$

where $u_{1},u_{2},\;and\;u_{3}$ are the control input.

In order to prevent the trajectory of the controlled system from being disclosed, based on the method discussed in Section 3.1, the disturbance term is used to protect the protected controlled system as described below:

(10) $$\left\{\begin{array}{c} {\dot{x}}_{1}=\left(y_{1}+l^{-1}\varphi \left(t\right)\right)\left(z_{1}+l^{-1}\varphi \left(t\right)\right)-a\left(x_{1}+l^{-1}\varphi \left(t\right)\right)+u_{1}\\ {\dot{y}}_{1}=\left(x_{1}+l^{-1}\varphi \left(t\right)\right)\left(z_{1}+l^{-1}\varphi \left(t\right)\right)-b\left(y_{1}+l^{-1}\varphi \left(t\right)\right)+u_{2}\\ {\dot{z}}_{1}=c\left(z_{1}+l^{-1}\varphi \left(t\right)\right)-d\left(y_{1}+l^{-1}\varphi \left(t\right)\right)-\left(x_{1}+l^{-1}\varphi \left(t\right)\right)\left(y_{1}+l^{-1}\varphi \left(t\right)\right)+u_{3} \end{array}\right.$$

Let the error system be

$$e_{1}=x_{1}-x,\;e_{2}=y_{1}-y,\;e_{3}=z_{1}-z,$$

So,

(11) $$\left\{\begin{array}{c} {\dot{e}}_{1}=\left(y_{1}+l^{-1}\varphi \left(t\right)\right)\left(z_{1}+l^{-1}\varphi \left(t\right)\right)-a\left(x_{1}+l^{-1}\varphi \left(t\right)\right)-yz+ax+u_{1}\\ {\dot{e}}_{2}=\left(x_{1}+l^{-1}\varphi \left(t\right)\right)\left(z_{1}+l^{-1}\varphi \left(t\right)\right)-b\left(y_{1}+l^{-1}\varphi \left(t\right)\right)-xz+by+u_{2}\\ {\dot{e}}_{3}=c\left(z_{1}+l^{-1}\varphi \left(t\right)\right)-d\left(y_{1}+l^{-1}\varphi \left(t\right)\right)-\left(x_{1}+l^{-1}\varphi \left(t\right)\right)\left(y_{1}+l^{-1}\varphi \left(t\right)\right)-cx+dy+xy+u_{3} \end{array}\right.$$

**Theorem** **1.** *When k>c, protected controlled system (10) can finite-time synchronize to system (2) under the following controller:*

$$u_{1}=yz-y_{1}z_{1}-\left(y_{1}+z_{1}\right)l^{-1}\varphi \left(t\right)-l^{-2}\varphi^{2}\left(t\right)+al^{-1}\varphi \left(t\right)-e_{1}^{\eta }$$

$$u_{2}=xz-x_{1}z_{1}-\left(x_{1}+z_{1}\right)l^{-1}\varphi \left(t\right)-l^{-2}\varphi^{2}\left(t\right)+bl^{-1}\varphi \left(t\right)-e_{2}^{\eta }$$

$$u_{3}=-ke_{3}+x_{1}y_{1}+\left(x_{1}+y_{1}\right)l^{-1}\varphi \left(t\right)+xy+l^{-2}\varphi^{2}\left(t\right)-\left(c-d\right)l^{-1}\varphi \left(t\right)-e_{3}^{\eta }$$

*and*

$$T=\frac{2}{1-\eta }\frac{1}{\Phi }\ln\left(1+\frac{\Phi \upsilon^{\frac{1-\eta }{2}}\left(0\right)}{2^{\frac{1+\eta }{2}}}\right).$$

**Proof** **of** **Theorem** **1.** Error system (11) is reorganized as

$$\left\{\begin{array}{c} {\dot{e}}_{1}=-ae_{1}+y_{1}z_{1}+\left(y_{1}+z_{1}\right)l^{-1}\varphi \left(t\right)+l^{-2}\varphi^{2}\left(t\right)-al^{-1}\varphi \left(t\right)-yz+u_{1}\\ {\dot{e}}_{2}=-be_{2}+x_{1}z_{1}+\left(x_{1}+z_{1}\right)l^{-1}\varphi \left(t\right)+l^{-2}\varphi^{2}\left(t\right)-bl^{-1}\varphi \left(t\right)-xz+u_{2}\\ {\dot{e}}_{3}=ce_{3}-de_{2}+\left(c-d\right)l^{-1}\varphi \left(t\right)-x_{1}y_{1}-\left(x_{1}+y_{1}\right)l^{-1}\varphi \left(t\right)-l^{-2}\varphi^{2}\left(t\right)+xy+u_{3} \end{array}\right.$$

Let $\upsilon \left(t\right)=\frac{1}{2}\left(e_{1}^{2}+e_{2}^{2}+e_{3}^{2}\right)$. Then, $\dot{\upsilon }=e_{1}{\dot{e}}_{1}+e_{2}{\dot{e}}_{2}+e_{3}{\dot{e}}_{3},$ so

$$\dot{\upsilon }=e_{1}\left(-ae_{1}+y_{1}z_{1}+\left(y_{1}+z_{1}\right)l^{-1}\varphi \left(t\right)+l^{-2}\varphi^{2}\left(t\right)-al^{-1}\varphi \left(t\right)-yz+u_{1}\right)+\;e_{2}\left(-be_{2}+x_{1}z_{1}+\left(x_{1}+z_{1}\right)l^{-1}\varphi \left(t\right)+l^{-2}\varphi^{2}\left(t\right)-bl^{-1}\varphi \left(t\right)-xz+u_{2}\right)+\;e_{3}\left(ce_{3}-de_{2}+\left(c-d\right)l^{-1}\varphi \left(t\right)-x_{1}y_{1}-\left(x_{1}+y_{1}\right)l^{-1}\varphi \left(t\right)-l^{-2}\varphi^{2}\left(t\right)+xy+u_{3}\right)\;=-ae_{1}^{2}-be_{2}^{2}-\left(k-c\right)e_{3}^{2}-de_{2}^{2}-\left(e_{1}^{1+\eta }+e_{2}^{1+\eta }+e_{3}^{1+\eta }\right)\;\leq -ae_{1}^{2}-be_{2}^{2}-\left(k-c\right)e_{3}^{2}-de_{2}^{2}-{\left(e_{1}^{2}+e_{2}^{2}+e_{3}^{2}\right)}^{\frac{1+\eta }{2}}\;\leq -\frac{1}{2}\left(e_{1}^{2}+e_{2}^{2}+e_{3}^{2}\right)\Phi -{\left(\frac{e_{1}^{2}+e_{2}^{2}+e_{3}^{2}}{2}\right)}^{\frac{1+\eta }{2}}2^{\frac{1+\eta }{2}}\;\leq -\Phi \upsilon \left(t\right)-2^{\frac{1+\eta }{2}}\upsilon^{\frac{1+\eta }{2}}\left(t\right)$$

where $\Phi =min\left\{a,b+d,k-c\right\}$. Based on Lemma 2, error system (11) is finite-time stable, and

$$T=\frac{2}{1-\eta }\frac{1}{\Phi }\ln\left(1+\frac{\Phi \upsilon^{\frac{1-\eta }{2}}\left(0\right)}{2^{\frac{1+\eta }{2}}}\right).$$

□

**Remark** **6.** *Finite-time control is a control technique based on the theory of finite-time stability, aimed at ensuring that the controlled system reaches a stable state within finite time. The basic principle is to compare the output signal of the controlled object with the expected value, obtain an error signal, and adjust the output of the controller based on this error signal, thereby achieving precise control of the controlled object.*

**Remark** **7.** *The financial dynamic system model helps us better understand the operating mechanism of financial markets by considering the interaction relationships between various components in the system. By simulating various scenarios in the financial market, the potential impact of systemic risks can be evaluated, and policy recommendations can be provided to management departments. However, the stability analysis and control of the financial system in this article can only provide a rough trend in system evolution and cannot accurately predict specific values. This is because the economic and financial system is influenced by many complex factors, such as external shocks, policy interventions, etc. The impact of these factors is difficult to fully capture and predict through mathematical models and requires comprehensive evaluation based on actual situations. Therefore, the financial model in this article has limitations. In particular, empirical analysis based on actual data will be our next research topic.*

### 3.3. A Simulation Example

**Figure 10:**
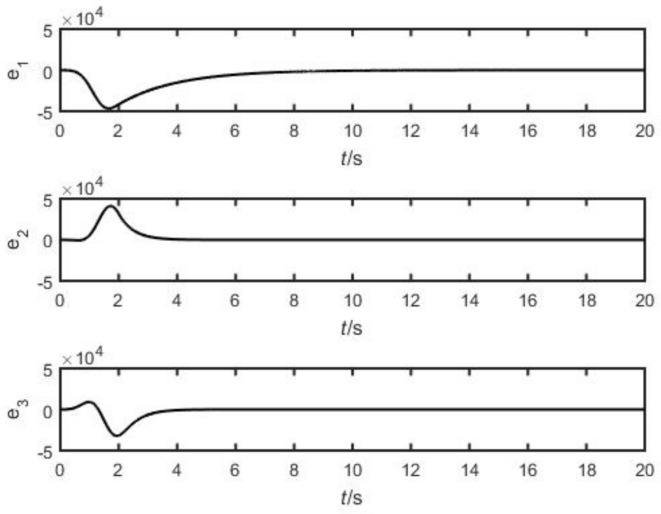
Synchronous error.

In simulation, let l=1,η=0.5, and k=5. The initial values of the two systems are (2, 3, 4) and (3, 4, 5), respectively. The synchronization error is depicted in Figure 10. Figure 10 shows that the synchronization error of the two systems gradually approaches 0 with time t; that is, the dynamic orbit of the drive system converges to the dynamic orbit of the response system, so that the two systems always keep pace in future time. The effectiveness of the designed method was verified through simulation.

## 4. Conclusions

This paper presents a novel dynamic model of financial risk contagion from financial principles, and it discusses several fundamental dynamic characteristics of the new chaotic system. Secondly, based on a perturbation-controlled system in finite time, a privacy protection method was designed. A finite-time controller was designed to realize system synchronization in finite time and protect the privacy of the controlled system synchronization process. The effectiveness of the proposed method was further validated by numerical simulation. Of course, the model in this article also has limitations, such as the fact that there are many factors that affect the financial market [9,28,29], and each factor has a different degree of impact, while the influencing factors considered in this article are slightly singular. In addition, the economic relationships hidden in financial data (such as regression, cointegration, causality, etc.) are an evolutionary process. The advantage of financial dynamical systems is that they maintain most of the dynamic properties of the original financial data and can explore the dynamic evolution process of financial data at the micro-level. Therefore, once the dynamic mechanism of this evolution is determined, it is of great significance for understanding and analyzing the evolutionary behavior of data.

## Data Availability

Data are contained within the article.
